# Effect of Acetyl Group on Mechanical Properties of Chitin/Chitosan Nanocrystal: A Molecular Dynamics Study

**DOI:** 10.3390/ijms17010061

**Published:** 2016-01-05

**Authors:** Junhe Cui, Zechuan Yu, Denvid Lau

**Affiliations:** 1 Department of Architecture and Civil Engineering, City University of Hong Kong, Hong Kong, China; junhecui2-c@my.cityu.edu.hk (J.C.); zechuanyu2-c@my.cityu.edu.hk (Z.Y.); 2 Department of Civil and Environmental Engineering, Massachusetts Institute of Technology, Cambridge, MA 02139, USA

**Keywords:** chitin, chitosan, acetyl group, fracture energy, stiffness, steered molecular dynamics (SMD) simulation

## Abstract

Chitin fiber is the load-bearing component in natural chitin-based materials. In these materials, chitin is always partially deacetylated to different levels, leading to diverse material properties. In order to understand how the acetyl group enhances the fracture resistance capability of chitin fiber, we constructed atomistic models of chitin with varied acetylation degree and analyzed the hydrogen bonding pattern, fracture, and stress-strain behavior of these models. We notice that the acetyl group can contribute to the formation of hydrogen bonds that can stabilize the crystalline structure. In addition, it is found that the specimen with a higher acetylation degree presents a greater resistance against fracture. This study describes the role of the functional group, acetyl groups, in crystalline chitin. Such information could provide preliminary understanding of nanomaterials when similar functional groups are encountered.

## 1. Introduction

Chitin, poly (*β*-(1→4)-*N*-acetyl-d-glucosamine), is a natural polysaccharide of major importance as universal template in biomineralization [1] as well as the main structural component of invertebrates skeletons [2]. Chitin-based biological materials attract a lot of research interest due to their huge sources in nature and excellent material and especially mechanical properties with broad variety of applications in bioinspired materials sciences and biomimetics [3]. It is widely known that chitin is the second most abundant substance on our planet ranked after cellulose and the most abundant nitrogen-bearing organic compound in nature [4]. Because of their outstanding mechanical properties, such as high strength, high toughness and being lightweight [5,6], chitin acts as a major load-bearing component in the exoskeleton of diatoms [7], sponges [8], corals [9] and mostly in the arthropod cuticles and shells of crustaceans (e.g., shrimps, crabs and lobsters) [10,11]. In these stiff tissues, chitin exists as fibers wrapped up by an extensive stabilized protein matrix which has a consistent interaction with chitin [12], together with modicum calcium carbonate [13], silica [14], even both mineral phases [15]. In order to achieve chitin for utilization purposes, a series of extraction process is adopted [16], including acid treatment to remove calcium carbonate, followed by alkaline treatment to remove proteins [4,12,17] and pigments [8,9,18]. In addition to the removal of protein, alkaline treatment can partially deacetylate chitin and produce a mixture of chitin and its most important derivative chitosan [17] in the solvent with pH value of about 9.71 [19]. Apart from the artificial deacetylation process, chitin is naturally partial deacetylated and natural chitin fiber is a composite system with different proportions of chitin and chitosan [20]. Compared with chitin in terms of chemical structure, chitosan only lacks acetyl group [21]. This little structural difference leads to significant distinctions between chitin and chitosan in many material properties (e.g., solubility, *etc.*) [4,22]. For instance, as a result of the hydrophobic property of acetyl group, chitin lacks solubility while chitosan is soluble in aqueous medium [4,23]. Likewise, it will logically bring about questions such as how acetyl group can affect the mechanical properties of chitin and chitosan and whether there exists a critical degree of deacetylation that distinguishes the mechanical behavior of chitin/chitosan. Therefore, attention should be paid to the effect of acetyl group in order to get the maximum potential of chitin/chitosan nanocrystal by manipulating the acetyl group.

In terms of mechanical properties, a lot of previous studies mainly focus on measuring Young’s modulus by performing experimental tensile tests [24,25] on chitin-based materials. Nevertheless, though many parts of cuticles, like insect wings, are subjected to high loads, which cause cuticles to fail by fracture, little has been carried out to investigate fracture properties of chitin based materials up to now [26]. Additionally, fracture and stress-strain behavior are the major factors governing the strength of a material and determining its application [27]. This indicates that a full-scale understanding of their mechanical properties is insufficient. Moreover, in order to unravel the underlying mechanisms governing these properties and make full use of chitin/chitosan nanocrystal, it is of utmost importance to examine the structure-properties relationship from the molecular level [28]. Once we have a thorough knowledge about principles of design and construction of composites by nature from the molecular level upwards [12], we can put it into practice and use nanofibers, resembling chitin, in composite design for improving mechanical properties [26,29]. At an atomistic level, molecular dynamics (MD) simulation, which investigates mechanical properties both systematically and comprehensively [5], can be used to explore the structure-property relations. In previous studies using MD simulation, stiffness and ductility along the chitin chain direction have been studied, revealing these properties are governed by covalent bonds [30]. In this paper, our objective is to investigate effect of acetyl group on mechanical properties of chitin/chitosan nanocrystal by examining fracture energy and stress-strain behavior along the directions, which are determined by much weaker non-bonded interactions (e.g., hydrogen bond and van der Waals interactions. To achieve this aim, we simulate the atomistic behavior of chitin nanocrystals subjected to fracture. Acetyl group contributes to the stabilizing of hydrogen bond (H-bond) network, increasing the resistance against fracture. Our findings will give inspiration for designing the nanomaterials with similar functional groups.

## 2. Results and Discussions

In this section, we present the analysis for the simulation aforementioned, including the nanostructure, fracture energy and stress-strain behavior of chitin/chitosan nanocrystal. Based on the results, we show our findings about the role of the acetyl group.

### 2.1. Structure of Chitin/Chitosan Nanocrystal

**Figure 1:**
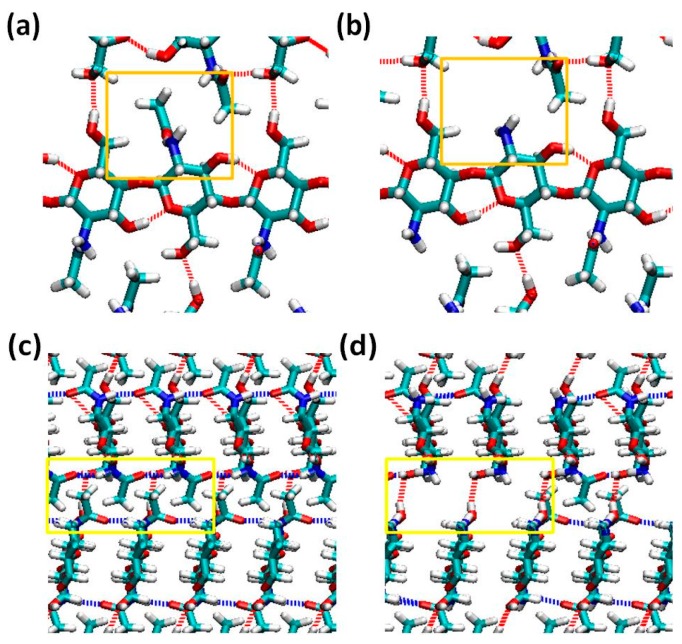
H-bonds formed within each model along the inter-chain direction (**a**,**b**) and inter-sheet direction (**c**,**d**). The orange and yellow boxes highlight the difference in H-bond pattern along inter-chain and inter-sheet directions respectively. The dashed lines stand for H-bonds and the color represents the acceptor atom. (**a**,**c**) are the H-bond formation for a chitin molecule; (**b**,**d**) are H-bond formed for a chitosan molecule. Comparing (**a**,**b**), we note that the acetyl group does not contribute to the formation of H-bond linking inter-chain direction. Comparing between (**c**,**d**), we note that the molecule without an acetyl group does not form the blue H-bonds linking inter-sheet direction.

**Figure 2:**
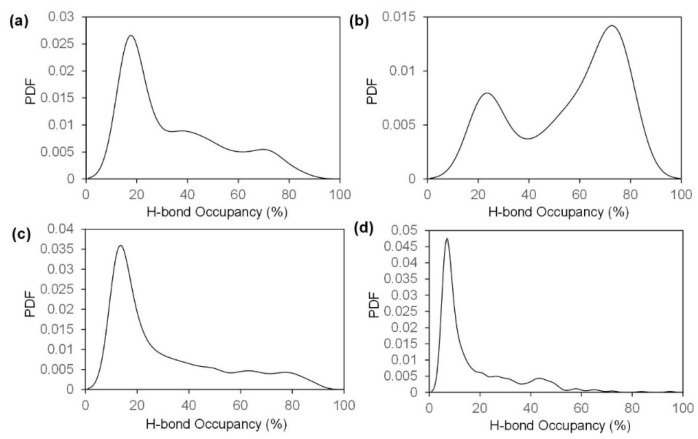
Probability density function (PDF) of H-bond occupancy for each pulling condition based on the last 600 ps of equilibrium simulation. Higher occupancy indicates a stable H-bond. (**a**,**b**) Pulling along inter-chain direction and inter-sheet direction in chitin model; (**c**,**d**). Pulling along inter-chain direction and inter-sheet direction in an 85% chitosan model.

We conduct steered molecular dynamics (SMD) simulations along the inter-chain and inter-sheet directions, where the mechanical properties are governed by non-bonded interactions that could be quantified by analyzing the H-bonding network through Visual Molecular Dynamics (VMD). As a result of this, we analyze the interaction within the nanocrystal by plotting the probability density function (PDF) for occupancy of the H-bond formed between different parts of the model under different pulling conditions. By definition, an H-bond is formed by an acceptor, a hydrogen atom and a donor [31]. We choose specific atoms (N, O, and F) as donors or accepters in H-bond detection by VMD and an H-bond is detected when the distance between acceptor and hydrogen is less than or equal to 4 Å and the angle between donor-hydrogen-acceptor is less than or equal to 35° [31,32]. Adopting these cut-offs, Figure 1 shows the H-bond formation for a single chitin molecule and chitosan molecule viewing from two perpendicular directions. H-bond occupancy is defined as the percentage of time that a hydrogen bond is considered “on” during the simulation [33], and it provides a measure for the strength of cohesion along a plane. For pulling along inter-chain direction, the occupancy of H-bonds formed between block 1 and block 2 and block 3 and block 4 are studied while H-bonds between block 1 and block 3 and block 2 and block 4 are analyzed for pulling along the other direction. The results of VMD analysis, which is about the occupancy of H-bonds within each model are plotted in Figure 2.

Based on the H-bond formation and PDF of H-bond occupancy, some reasonable comparisons could be made. As shown in Figure 1, some H-bonds linking the inter-sheet direction are refrained in chitosan model as a result of the absence of acetyl group. Previous studies have demonstrated that the distribution of acetyl group along the chain direction contributes to the H-bond formation in chitin model [4,34]. Our analysis results coincide well with this conclusion. The occupancy of H-bonds formed along the inter-chain direction in the chitin model are concentrated at about 20%, and the frequency of high occupancy (more than 70%) H-bonds is very low. On the contrary, most of the H-bonds formed along the inter-sheet direction have an occupancy of 65% to 90% while the frequency of low occupancy H-bonds is only about 1/3 that of high occupancy ones. This distinction in terms of H-bond occupancy will result in disparate mechanical properties (e.g., fracture energy and stress-strain curve) along the two separate directions. However, this contrast does not apply to the 85% chitosan model, whose H-bond occupancy distribution is similar along both of the two directions, and most of its H-bonds are concentrated at a low occupancy region. Apart from H-bonds, another significant component of non-bonded interaction is van der Waals interaction [35], which depends largely on the molecular mass. The molecular mass of chitin molecules are apparently larger than that of chitosan molecules because the acetyl group is greater in mass than a single hydrogen atom. The distinction in terms of non-bonded interaction between chitin and chitosan model will result in diverse mechanical properties.

### 2.2. Fracture and Stress-Strain Behavior

**Figure 3:**
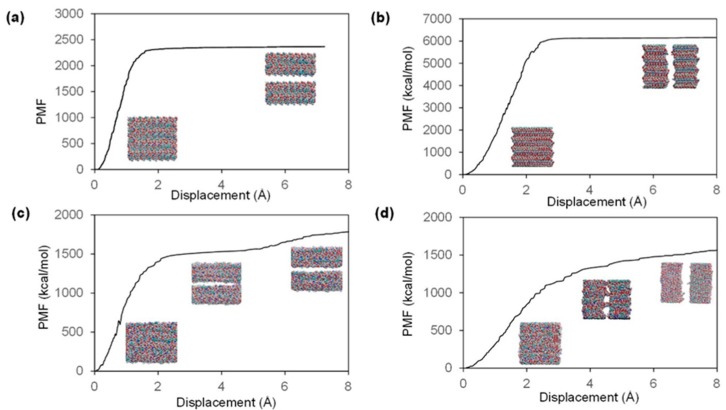
PMF profiles for each pulling scenario. The “Savitzky–Golay filter” method is adopted to draw a smooth curve by fitting a cubic function for every 100 raw data points. The VMD snapshots show the corresponding pulling situations. (**a**,**b**) are PMF profiles for chitin model pulling along inter-chain and inter-sheet directions respectively; (**c**,**d**) are PMF profiles for 85% chitosan model pulling along inter-chain and inter-sheet directions, respectively.

Based on the value of potential of mean force (PMF) computed by SMD simulation, the PMF profiles (Figure 3), also known as one dimensional free energy of chitin/chitosan nanocrystal [36], are plotted for two pulling directions of both models. The maximum PMF for chitin model along the inter-chain direction is about 2350 kcal/mol, which is much smaller than that along the inter-sheet direction (6135 kcal/mol). The fracture energy is calculated to be 0.33 $\text{kcal}/\left(\text{mol}\cdot {Å}^{2}\right)$ and 0.86 $\text{kcal}/\left(\text{mol}\cdot {Å}^{2}\right)$ for the inter-chain and inter-sheet directions separately. According to this data, the chitin nanocrystal is a very brittle material because the fracture energy is much less than typical brittle material—glass (3 to 5 $\text{kcal}/\left(\text{mol}\cdot {Å}^{2}\right)$) [37,38]. Since there is no covalent bond formed along the two pulling directions, the causes for the differences in fracture energy stem from distinctions in non-bonded interactions [38,39]. From the PMF profile shown in Figure 3c,d, the maximum PMF of 85% chitosan model is estimated to be 1600 kcal/mol for both inter-chain direction and inter-sheet direction. The corresponding values for fracture energy are computed to 0.29 $\text{kcal}/\left(\text{mol}\cdot {Å}^{2}\right)$. Compared with pure chitin model, the 85% chitosan model presents a smaller fracture energy (approximately 25%–65%) along both pulling directions. Moreover, for the crystalline chitin model, the PMF demonstrates a constant value when the two parts detach from each other. In contrast, after the amorphous chitosan model is separated into two major parts, the value of PMF is increasing, but much slower than before separation, because there are still some linkages between the two parts, which can be observed from the corresponding trajectory.

**Figure 4:**
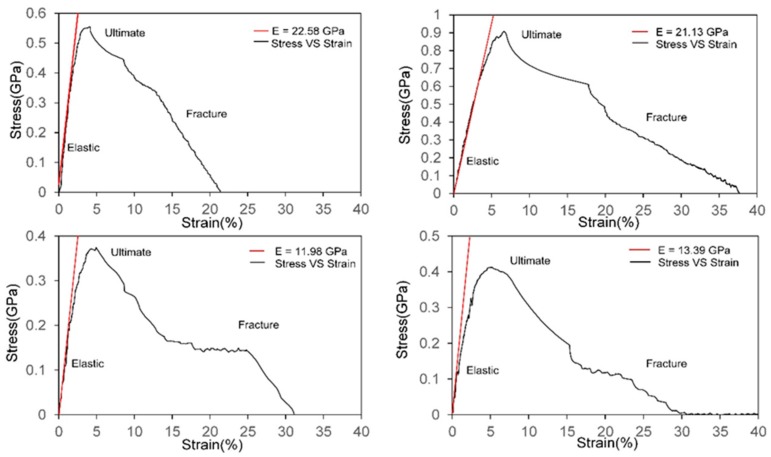
Stress-strain curves. The curves are initial stress-strain curves generated by the Savitzky–Golay filter method on the basis of the huge amount of raw data points. To generate a smooth curve correctly representing the relationship between stress and strain, we use a cubic function to fit for every 50 data points. The red lines are linear fit for the elastic region, and its slope represents the stiffness. The value of stiffness is shown in the legend. (**a**,**b**) are stress-strain curves for chitin model pulling along inter-chain and inter-sheet directions, respectively; (**c**,**d**) are stress-curves for 85% chitosan model pulling along inter-chain and inter-sheet directions respectively.

We can estimate the elastic constant and stress-strain behavior of chitin/chitosan nanocrystal according to the output values of pulling force. The stress acted on the nanocrystal is estimated according to method previously mentioned. Figure 4 illustrates the stress-strain curve along each pulling direction for both chitin and 85% chitosan models. For all those curves, the stress along the pulling direction shows a linear elastic response before reaching the maximum value. The elastic constant, or equivalently stiffness, together with corresponding ultimate stress and fracture strain, can be estimated from the stress-strain curves plotted and the values are listed in Table 1.

**Table 1:** Summary of stress-Strain behavior.

Model	Direction	Ultimate Stress (GPa)	Fracture Strain (%)	Stiffness (GPa)
Chitin	Inter-chain	0.551	5.29	22.58
	Inter-sheet	0.936	7.45	21.13
85% Chitosan	Inter-chain	0.372	6.22	13.39
	Inter-sheet	0.419	8.82	11.98

The elastic constants of the chitin model are 22.58 and 21.13 GPa for inter-chain and inter-sheet directions distinctively. The two values of stiffness are more or less similar instead of a sharp contrast as in case of the fracture energy. The simulation results are nearly identical to the experimental measured results from previous studies [40], indicating our model making techniques and simulation methods are suitable for the studying purpose. However, the stiffness value is much lower than the simulation result by pulling along the chitin chain direction (92.26 GPa) [30,41] because inter-chain and inter-sheet directions are stabilized by much weaker interactions instead of strong covalent bonds. Furthermore, the ultimate stress and fracture strain for pulling along the inter-sheet direction are much larger than that along the inter-chain direction.

However, for the chitosan model, the ultimate stress along inter-sheet direction is slightly instead of significantly larger than that of inter-chain direction, which conforms the H-bond occupancy distribution previously mentioned. When compared with the chitin model, more distinctions can be detected. From Table 1, the stiffness and ultimate stress of the 85% chitosan model along both pulling directions are much smaller than the corresponding values of chitin model, indicating that the chitosan model is less stiff. In contrast, the fracture strains are almost 1.3 times that of the chitin model, revealing that the chitosan model is more ductile than the chitin model.

### 2.3. The Role of Acetyl Group

The only chemical difference between chitin and chitosan is the acetyl group. The presence of the acetyl group causes more high occupancy H-bonds along the inter-sheet direction of chitin model. In contrast, as there are few acetyl groups within the chitosan model, the H-bond occupancy along two directions is similar. Additionally, van der Waals interaction within chitin crystals is significantly enlarged due to the larger molecular mass of acetyl group. The effect of acetyl group on these non-bonded interactions results in distinct mechanical properties between chitin and chitosan.

From the H-bonding network aforementioned, H-bond occupancy is correlated to strength of cohesion along a plane. An H-bond with higher occupancy leads to greater fracture strength according to stochastic theory of fracture [33,38]. Within the chitin model, acetyl groups contribute to the formation of stable H-bonds (higher occupancy) along the inter-sheet direction. Fracture along this direction requires more energy to break these high occupancy H-bonds. It is the presence of acetyl group that leads to much higher fracture energy and larger ultimate stress along the inter-sheet direction. Meanwhile, the relatively smaller fracture energy and smaller ultimate stress in the inter-chain direction indicate a higher possibility for the chitin crystal to fail by fracture between different chitin layers under high loading conditions. In contrast with the chitin model, there is no significant distinction in terms of fracture and stress-strain behavior along two pulling directions within chitosan model due to lack of acetyl group. Moreover, during the pulling process, there are some linkages between two parts of chitosan model instead of a thorough separation. This phenomenon is correlated to the amorphous nature of chitosan model resulted from the lack of acetyl group. Additionally, the fracture energy and ultimate stress of chitosan model is smaller than that of chitin model due to little contribution of acetyl group on increasing the number of H-bond and van der Waals interaction. However, without the supporting effect of the acetyl group, the atoms are in much closer contact in the amorphous chitosan model. The closer molecular contact leads to a shorter original length along the pulling direction as well as a larger deformation before fracture occurs. As a result of this, the chitosan model demonstrates a better performance in terms of ductility. To summarize, the presence of the acetyl group demonstrates the following effects on mechanical properties of chitin/chitosan nanocrystal: increasing the resistance against fracture while decreasing the ductility by affecting the non-bonded interactions. In the industrial field, materials of different functions require various mechanical properties. In some conditions, higher fracture energy material is required while ductile material is preferred in other conditions. The findings in this paper will inspire us to obtain the most appropriate mechanical properties and fully utilize chitin-based materials by adjusting the acetyl group.

## 3. Methods

In this paper, we have constructed two models. The first one is pure chitin model and the second one is with an 85% degree of deacetylation (*i.e.*, 85% chitosan and 15% chitin). It is practical to extract 85% deacetylated chitosan from natural sources [42]. Many previous studies adopted 85% chitosan as the high degree of deacetylation sample [43,44,45]. Therefore, we choose the 85% chitosan model to form a sharp contrast with the pure chitin model so that the effect of the acetyl group is apparent. In addition, these two models can reflect the degree of deacetylation of real chitin/chitosan samples. Steered molecular dynamics (SMD) simulation has been proven as a good way for investigating fracture behavior in previous measurements [46,47,48,49]. In each model, SMD simulations are performed along both inter-chain and inter-sheet directions separately. The model making techniques and simulation details will be discussed in the following subsections.

### 3.1. Atomistic Models

**Figure 5:**
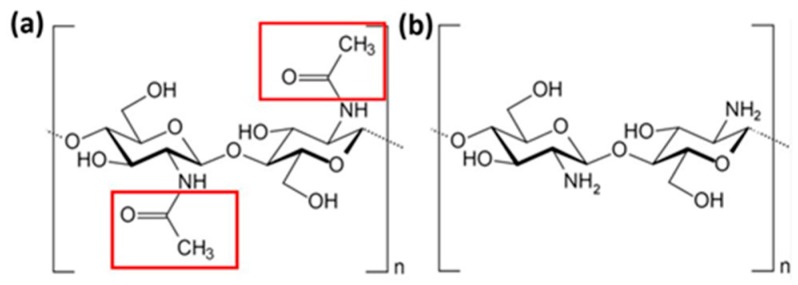
(**a**) Chemical structure of chitin molecule; (**b**) chemical structure of chitosan. The acetyl group is highlighted in the red box.

**Figure 6:**
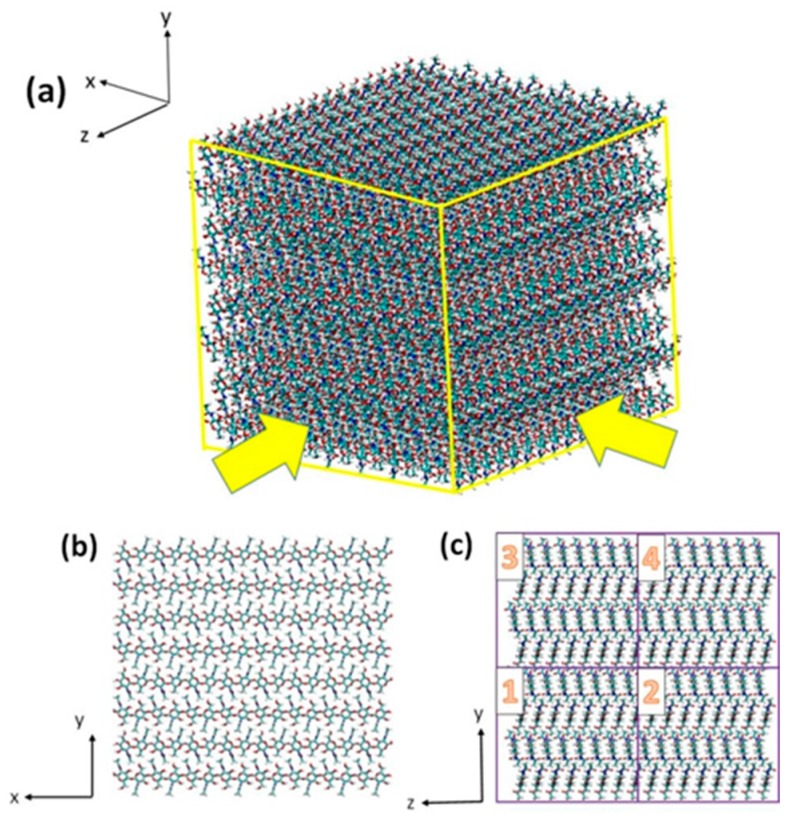
(**a**) The 3D view of the complete chitin model; (**b**,**c**) Two cross sectional views of chitin model (**c**) also shows the model numbering system. Numbers 1–4 correspond to the four regions divided to make atom-selection easier for later SMD simulation work.

Chitin polymer consists of a linear chain of poly *β*-(1→4)-*N*-acetyl-d-glucosamine [21]. Figure 5a shows the chemical structure of chitin molecule. In nature, chitin exists as three kinds of crystal forms, namely *α*-chitin, *β*-chitin and *γ*-chitin. Among them, the most abundant crystal form is *α*-chitin [50], which has a structure of antiparallel chains. Previous studies have revealed the structure of its unit cell [19,51], which have been used by us to construct our chitin model. We divide the whole model into four regions in the first place in order to make atom selection easier for future simulation work. The *x*, *y*, and *z* axes in the picture correspond to the <100>, <010> and <001> directions, respectively. The whole model contains 55,680 atoms in total and the dimension of the model is 84.51 × 77.02 × 82.25 Å, which is an optimum size for studying of fracture behavior [38]. Figure 6 shows the final layout and numbering system of this model.

**Figure 7:**
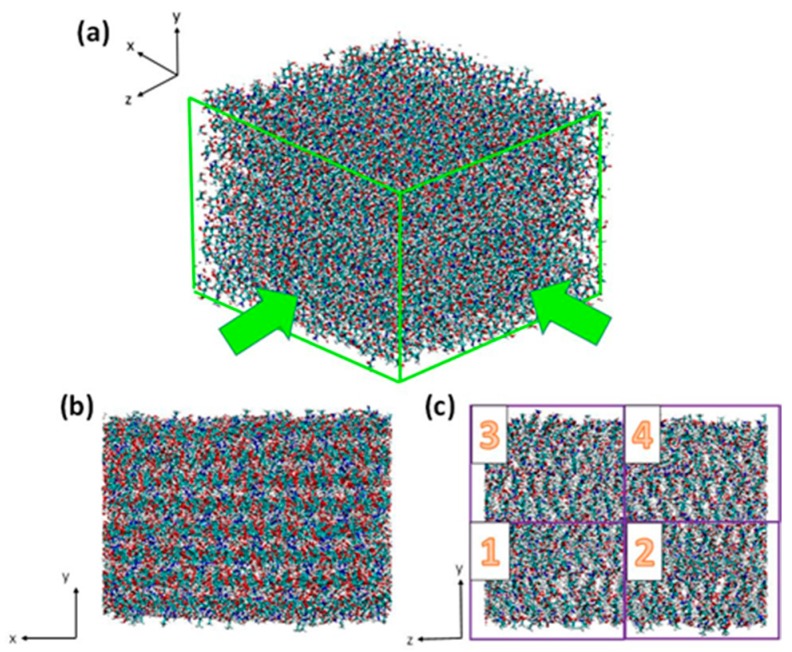
(**a**) The 3D view of the complete chitosan model; (**b**,**c**) Two cross sectional views of chitosan model. (**c**) Model numbering system. Numbers 1–4 correspond to the four regions divided to make atom-selection easier for later SMD simulation work. In comparison with the crystalline structure of chitin, this amorphous structure presents a closer molecular contact, revealing the unstable nature of highly deacetylated chitosan.

Chitosan is either an artificially or naturally deacetylated derivative of chitin which is endogenously produced in vertabrates [52]. It is with linearity in arrangement and composed of *β*-(1→4)-linked-d-glucosamine [53]. Meanwhile, as is shown in Figure 5b, we can see its chemical structure. As the deacetylated derivative of chitin, chitosan exists together with chitin in chitin/chitosan nanocrystals with different degrees of deacetylation. As previously mentioned, one of the frequently used experimental samples is 85% deacelytated chitosan [42,43,54]. Moreover, chitin crystal will lose its crystallinity during the transformation process to chitosan, resulting in an amorphous shape of the extracted chitosan [43,55]. Therefore, our second model is constructed by replacing 85% chitin molecules by chitosan molecules randomly and evenly. In order to build the model in highly conformity with reality, we design it non-symmetrically in any direction (Figure 7). Moreover, this partially deacetylated model contains 47,040 atoms.

### 3.2. Simulation Details

In this study, Large-scale Atomic/Molecular Massively Parallel Simulator (LAMMPS) is used to do simulations for the two chitin/chitosan nanocrystal models. We use CHARMM36 force fields for chitin and chitosan molecules throughout the whole simulation process [56]. A cutoff of 10 Å (10^−10^ m) is adopted for the non-bonded interactions. We use the particle-particle particle-mesh (PPPM) method to compute the long-range Columbic interactions. The SHAKE algorithm is used to constraint high-frequency dynamics from hydrogen-related energy terms [57]. Periodic boundary condition is set in all the <100>, <010> and <001> directions but along the <010> direction, sufficient space between the mirror images is reserved for future simulation work. The above settings were adopted in the past research works about mechanical properties of chitin-based material [19,28,30]. Similarly, we also adopt the method from previous studies for the equilibrium simulation process. Firstly, the system is minimized to reach a minimum-energy status by adjusting the atom coordinates according to conjugate gradient algorithm. The system is then heated up from 50 to 300 K in 20 ps. After that, it is equilibrated at 300 K for 50 ps in a canonical (NVT) ensemble (amount of substance/volume /temperature remain constant) followed by equilibration at 300 K and 1 atm for 500 ps in an isothermal-isobaric (NPT) ensemble (amount of substance/pressure/temperature remain constant). The temperature control is achieved using Nose-Hoover thermalstat. Finally, the system is equilibrated in an NVT ensemble for another 1 ns and the corresponding output file is saved for later simulations. The models have achieved equilibrium state because the computed root mean square of deviation (RMSD) value has become constant.

**Figure 8:**
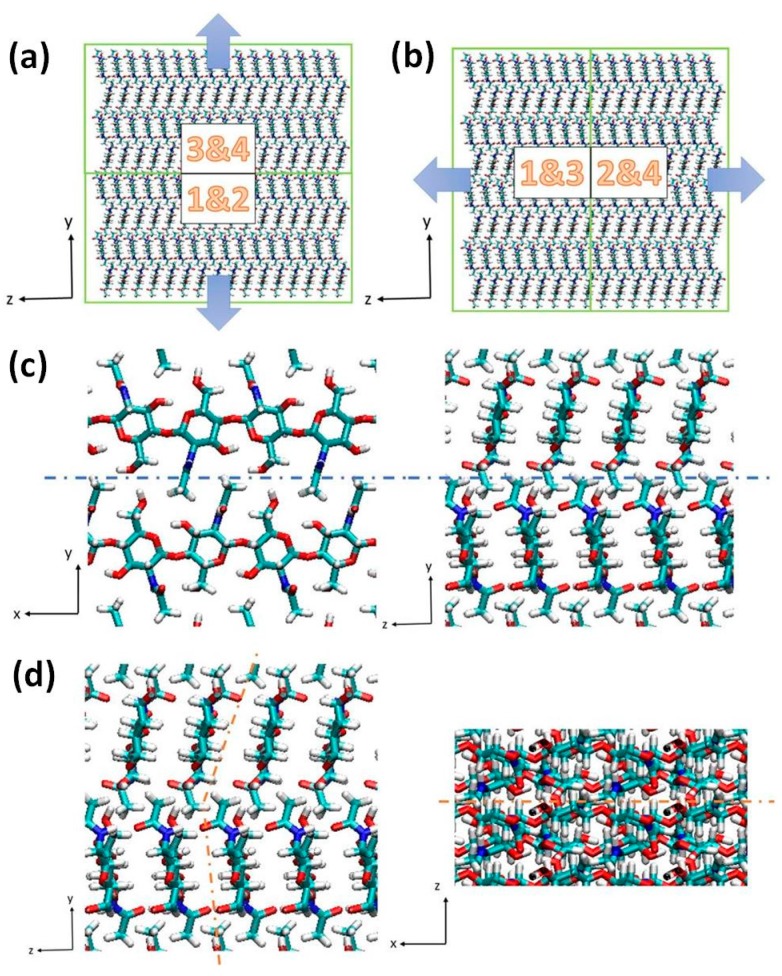
Two sets of SMD simulation. The green blocks represent different parts of the model and the numbers demonstrate the combination of the four parts divided previously. The blue arrows demonstrate the direction of pulling force. (**a**) Pulling along the inter-chain direction; (**b**) Pulling along inter-sheet direction; (**c**,**d**) illustrate the inter-chain and inter-sheet directions, where the dotted blue and orange lines correspond to the inter-chain and inter-sheet interactions respectively.

After equilibrium simulation, we perform steered molecular dynamics (SMD) simulation on the two models. During the simulation process, SMD force is applied by tethering one end of a virtual spring to a target atom while the other end is used for pulling. Two sets of SMD simulation are done on each model along two perpendicular directions as is shown in Figure 8. For the pulling along inter-chain (<010>) direction, the center of mass of block 3 and block 4 is chosen as a target atom tethered to the virtual spring while the center of mass of block 2 and block 4 is selected for simulation along the inter-sheet (<001>) direction. The momentum of the other half of the model is reset to zero. SMD in the constant speed mode is used to stretch the model and this SMD approach has been successfully used in previous similar studies [30,38]. Similar to previous research studying mechanical properties of cellulose, we adopt the same parameters in SMD simulation with speed of v=20 Å/ns and virtual spring constant of 100 kcal/(mol·Å^2^), respectively [38].

The trajectory of equilibrium simulation is used to analyze the H-bonding system within the models. From previous studies, the cut-off of donor-acceptor distance and donor-hydrogen acceptor angle are set to 4 Å and 35°, respectively, for detecting hydrogen bonds [19,28]. In the later 600 ps of equilibrium simulation, the information of H-bond is collected every 10 ps by VMD. The occupancy of H-bond over the last 600 ps is analyzed by means of plotting its probability density function (PDF) using MATLAB. During the SMD simulation process, LAMMPS makes a record every 100 fs for several useful values, namely potential of mean force (PMF), distance between center of mass (CM) of two half models and spring force along the pulling direction. It is very significant to record these values for analyzing the simulation results in the later stage. We use potential of mean force (PMF) to represent work done by the virtual spring during the SMD simulation process [58]. We calculate the displacement of the end of virtual spring *x*(*t*) and model strain (σ) along the pulling direction using the distance between CMs of the pulling part and stable part of the model. By plotting PMF against displacement of spring end, we can generate PMF profile of which the maximum constant value can be used to compute the fracture energy. The stress applied by the virtual spring is estimated from the force applied along the pulling direction. Based on the values calculated above, stress-strain curve is plotted and the stiffness is estimated as the slope of this curve in the linear elastic range.

## 4. Conclusions

In this research, the effects of acetyl group on two mechanical properties—fracture energy and stress-strain behavior—are studied due to the load-bearing function of chitin fibers in natural chitin-based materials. We construct two typical atomistic models, namely pure chitin model and 85% chitosan model. The chitosan model is built amorphously to fit the realistic structure of chitosan, which has not been taken into account in previous molecular dynamics studies. In each model, we performed two sets of steered molecular dynamics (SMD) simulations along inter-sheet direction and inter-chain direction, respectively, to measure the fracture energy and study the stress-strain behavior. For the chitin model, the fracture energy for pulling along inter-chain direction is lower, indicating the fracture is more likely to occur along this direction. The fracture energy of 85% chitosan model is about 25% to 65% of chitin model, and this difference is the result of stronger non-bonded interactions within the chitin model due to the presence of the acetyl group. The stiffness of chitin model is estimated to be 21.13 to 22.58 GPa, which is identical to experimental results. In contrast, the 85% chitosan model has a lower stiffness but larger strain, revealing the effect of acetyl group on increasing stiffness and decreasing ductility. Our findings could provide preliminary understanding of nanomaterials with acetyl groups and could further inspire us to design composite materials by appropriately introducing the acetyl group to achieve better mechanical performance. Our models are limited to demonstrating the mechanical behaviors at the atomistic level. In reality, materials exhibit diverse mechanical properties at different length scales. This study focuses on an atomistic investigation, and questions about acetyl groups at a large length scale remain unanswered. Future studies could adopt coarse-grained models and establish multiscale understanding of chitin-based materials.
